# Surgical Treatment for Thrombus Straddling a Patent Foramen Ovale

**DOI:** 10.5935/1678-9741.20160082

**Published:** 2016

**Authors:** Marcos Aurélio Barboza de Oliveira, Amália Tieco da Rocha Sabbag, Antônio Carlos Brandi, Carlos Alberto dos Santos, Paulo Henrique Husseni Botelho, Franz Andrei Patriarcha, Domingo M. Braile

**Affiliations:** 1 Faculdade de Medicina de São José do Rio Preto (FAMERP), São José do Rio Preto, SP, Brazil; Centro Universitário de Votuporanga (UNIFEV), Votuporanga, SP, Brazil.; 2 Hospital de Base (HB), São José do Rio Preto, SP, Brazil.; 3 Faculdade de Medicina de São José do Rio Preto (FAMERP), São José do Rio Preto, SP, Brazil.; 4 Faculdade de Medicina de São José do Rio Preto (FAMERP), São José do Rio Preto, SP, Brazil and Universidade de Campinas (UNICAMP), Campinas, SP, Brazil.

**Keywords:** Embolism, Paradoxical, Venous Thrombosis, Stroke, Pulmonary Embolism

## Abstract

We present a case of a 41-year-old female with deep vein thrombosis after abdominal surgery. The patient quickly developed severe pulmonary embolism and stroke representative of paradoxical embolism. Echocardiography showed a thrombus straddling a patent foramen ovale, which was confirmed intraoperatively. An accurate diagnosis and rapid treatment decisions are crucial for preventing patient deterioration in the form of new pulmonary embolisms or stroke.

**Table:** 

Abbreviations, acronyms & symbols	
**AP**	**=Arterial pressure**
**CT**	**=Computed tomography**
**CVP**	**=Central venous pressure**
**DVT**	**=Deep vein thrombosis**
**ETCO_2_**	**=End-tidal CO_2_**
**ICU**	**=Intensive care unit**
**FiO_2_**	**=Fraction of inspired oxygen**
**PE**	**=Pulmonary embolism**
**PFO**	**=Patent foramen ovale**

## INTRODUCTION

The identification of a thrombus in a patent foramen ovale (PFO) is a relatively rare event^[1-4]^, despite the relative frequency of this anatomical anomaly of the atrial septum, which can be found in almost 20% of the normal population^[1]^. Generally, thrombus straddling a PFO is a complication of severe thromboembolic disease and is a consequence of thrombus migration into the left heart chambers, which itself is due to pulmonary hypertension. Its association with massive pulmonary embolism (PE) leads to worse prognosis^[1]^.

Treatment options for thrombus in PFO include thrombolysis, heparin therapy, or surgery. Fauveau et al.^[2]^ reported a list of 88 cases in the literature; they found a mortality rate of 36% when thrombolysis was used, 14% when heparin was administered, and 13% when surgical treatment was employed. We report a case of a straddling thrombus in a patient with a PFO that was associated with massive PE and treated surgically.

## CASE REPORT

A 41-year-old female patient who was previously healthy had undergone a laparoscopic cholecystectomy ten days prior. Nine days after the procedure, the patient developed dysarthria and acute respiratory distress, requiring supplementary oxygen therapy via oxygen mask. Clinically, the patient presented left calf tenderness. Doppler ultrasound revealed lower limb venous thrombosis. Brain computed tomography (CT) showed no significant change; a chest CT confirmed the existence of an atrial thrombus straddling a PFO.

**Fig. 1:**
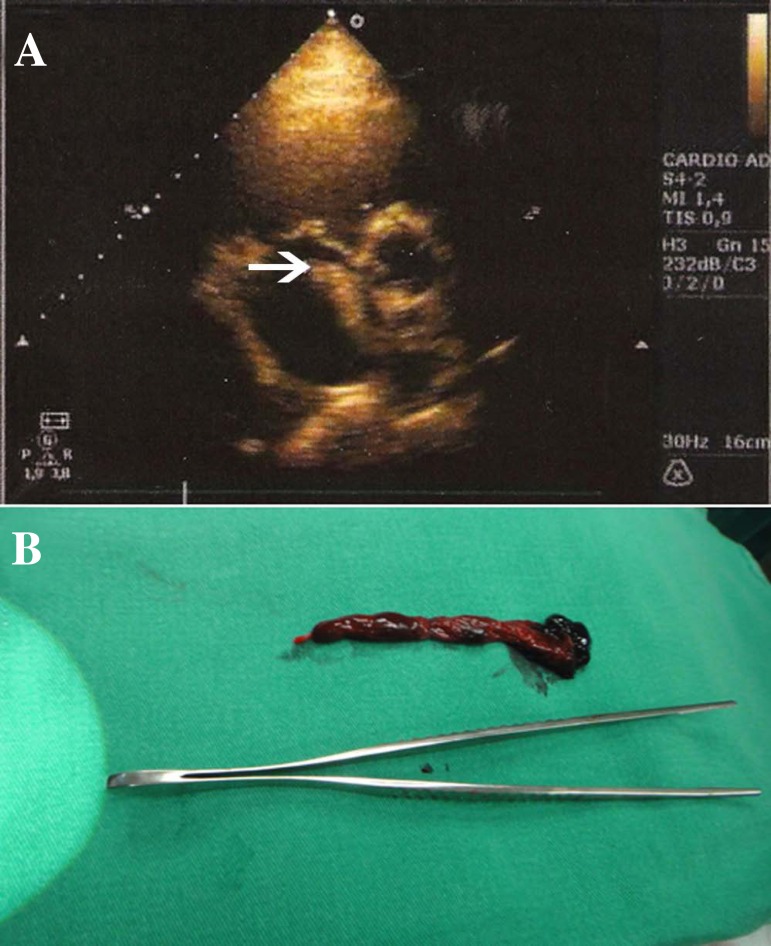
(A) Transthoracic echocardiogram showing thrombus straddling the patent foramen ovale (white arrow). (B) Thrombus approximately 5 cm in size removed from the foramen ovale.

Transthoracic echocardiogram revealed systolic pressure of the right ventricle to be 56 mmHg, as well as moderate tricuspid regurgitation secondary to pulmonary hypertension and a thrombus straddling a PFO (Figure 1A). Surgical treatment was indicated to remove the thrombus from the heart and for PFO closure. Figure 1B shows the thrombus removed from the atrium during the surgical intervention.

**Fig. 2:**
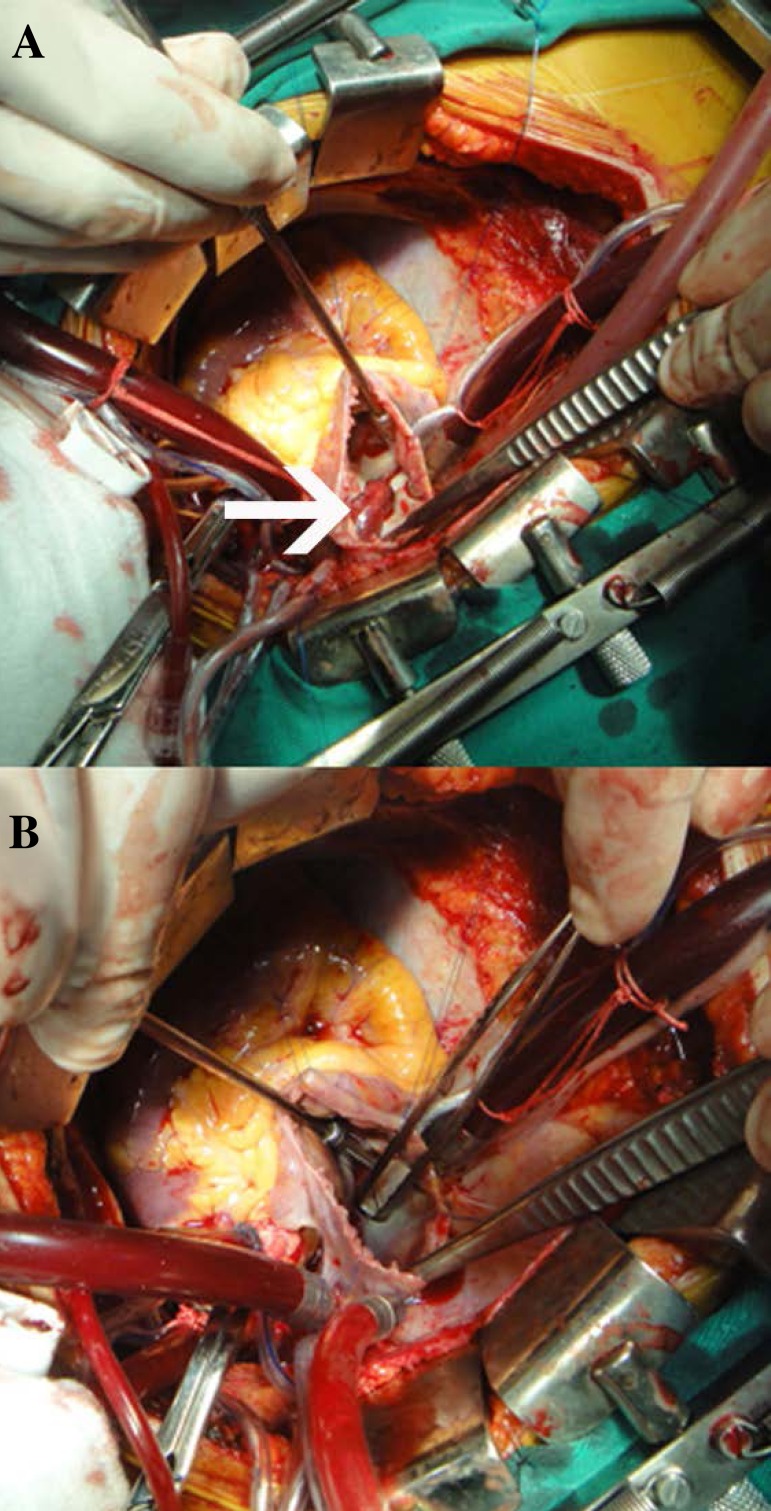
(A) Surgical picture showing open right atrium and thrombus in the patent foramen ovale (white arrow). (B) Tissue forceps through the patent foramen ovale, now without thrombus.

During the surgery, the manipulation of the heart was avoided in order to minimize the risk of another PE. The thrombus was found in the right atrium and it went through the PFO into the left atrium (Figures 2A and 2B). The thrombus was resected and PFO was closed using a 4-0 Prolene running suture.

Cardiopulmonary bypass weaning failed twice. Left atrial pressure was 30 mmHg, end-tidal CO_2_ (ETCO_2_) was 10-12 mmHg, and oxygen saturation was 89% (a value likely due to the PE). Norepinephrine (0.2 mcg/kg/min) and milrinone (0.75 mcg/kg/min) were useful for successfully weaning the patient off ventricular assistance.

The patient was admitted to the intensive care unit (ICU) under mechanical ventilation with a fraction of inspired oxygen (FiO_2_) of 60%, saturation at 89%, central venous pressure (CVP) of 24 mmHg, arterial pressure (AP) of 80x40, norepinephrine at 0.7 mcg/kg/min, and milrinone 0.75 mcg/kg/min. She remained unstable overnight but gradually exhibited improvements in hemodynamic stability, CVP reduction, and improved oxygen saturation in the days following. She was extubated on the sixth postoperative day and discharged from the ICU on the eighth postoperative day.

The present study was approved by Ethics Committee under number 1.401.186.

## DISCUSSION

Deep vein thrombosis (DVT) is the third largest cardiovascular disease in developed countries, followed by myocardial infarction and stroke. The risk factors for developing DVT include recent surgery and hormonal contraceptive therapy; DVT is also more common in women. These risk factors increase the likelihood of clot formation in the pelvis and lower limbs, which can eventually cause PE^[5]^.

The foramen ovale is closed by a flap-like valve on the left side of the left atrium. In patients with acute cor pulmonale, higher right-sided pressure may induce a reopening of the foramen ovale and a right-to-left atrial shunt or an increase in the shunt in patients with a PFO^[1]^. Most venous thrombi pass through the inferior vena cava and flow directly through the PFO, subsequently reaching the left atrium.

Reports of thrombus straddling the PFO are rare^[1-4]^. Guffi^[6]^ demonstrated the efficacy of the surgical closure of PFO as prophylaxis to prevent cryptogenic strokes. However, in Guffi's study, no patients were found to have thrombosis on the edges of the PFO, as was the case in our patient.

Generally, the diagnosis of paradoxical embolism is presumed^[6]^. Tests such as D-dimer, venous Doppler of lower limbs, and chest CTs are useful for the study of DVT and PE^[5]^, but when stroke is concurrent, brain CT and echocardiogram are mandatory^[2]^. In our patient, D-dimer was not performed because the initial symptoms were dyspnea and dysarthria. Therefore, chest and brain CTs were selected.

Although heparin therapy has been associated with the same mortality rate as surgical treatment^[2]^, and though PFO closure has not been associated with a reduction in cases of late recurrent stroke^[7]^, the presence of an intracardiac thrombus increases the risk of another acute PE and stroke, risks which justify emergency surgery for the intracardiac thrombus^[1,8]^.

Aggressive treatment should not be delayed. Surgical outcomes are more promising in stable patients without cardiogenic shock or massive systemic embolism. It should also be pointed out that most paradoxical embolisms occur early; they typically occur at the time of PE, as in our patient, and are likely caused by the increase in right atrial pressure^[1]^.

We have presented the case of a patient with PE and stroke in the early postoperative period following abdominal surgery. It is important to emphasize the need for full diagnostic investigations and swift decisions regarding treatment in order to avoid the worsening of symptoms in the form of new pulmonary embolisms or stroke.

**Table:** 

Authors’ roles & responsibilities	
MABO	Analysis and/or data interpretation; conception and design study; manuscript redaction or critical review of its content; realization of operations and/or trials; final manuscript approval
ATRS	Manuscript redaction or critical review of its content; final manuscript approval
ACB	Conception and design study; realization of operations and/ or trials; final manuscript approval
CAS	Manuscript redaction or critical review of its content; final manuscript approval
PHHB	Conception and design study; final manuscript approval
FAP	Manuscript redaction or critical review of its content; final manuscript approval
DMB	Analysis and/or data interpretation; final manuscript approval
